# Optimization-based design of an elastostatic cloaking device

**DOI:** 10.1038/s41598-018-28069-7

**Published:** 2018-06-29

**Authors:** Víctor D. Fachinotti, Ignacio Peralta, Alejandro E. Albanesi

**Affiliations:** 1 Centro de Investigación de Métodos Computacionales (CIMEC), Universidad Nacional del Litoral (UNL)/Consejo Nacional de Investigaciones Científicas y Técnicas (CONICET), Predio CCT-CONICET Santa Fe, Ruta 168, Paraje El Pozo, 3000 Santa Fe, Argentina; 2 Universidad Tecnológica Nacional (UTN)/Facultad Regional Santa Fe, Lavaisse 610, 3000 Santa Fe, Argentina

## Abstract

We present a new method for the design of devices to manipulate the displacement field in Elastic materials. It consists of solving a nonlinear optimization problem where the objective function defines the error in matching a desired displacement field, and the design variables determine the required material distribution within the device. In order to facilitate fabrication, the material at a given point of the device is chosen from a set of predefined materials, giving raise to a discrete optimization problem that is converted into a continuous one using the Discrete Material Optimization technique. The candidate materials maybe simple, isotropic materials, but the device made of them behaves as a whole as a metamaterial, enabling the manipulation of the displacement field in ways that are inconceivable in nature. As an example of application, a device for elastostatic cloaking or unfeelability is designed.

## Introduction

Cloaking, that is hiding objects to certain fields, was first considered by Hashin and Shtrikman^1^, who found that spheres with an appropriate coating do not disturb the magnetic flow in the surrounding material.

In 2006, simultaneously and independently, Leonhardt^2^ and Pendry *et al*.^3^ introduced the use of conformal mapping to determine the inhomogeneous and anisotropic refractive index for electromagnetic cloaking, giving birth to the approach based on transformation optics for metamaterial design. The inhomogeneity and the anisotropy of the cloaking medium are the result of applying the conformal mapping to a homogeneous medium under uniform electromagnetic field. The required materials may have extreme properties (for instance, negative refraction index). These properties are usually unconceivable in nature, and a composite has to be designed to mimic them; this is the so-called metamaterial.

Beyond electromagnetism, transformation optics was used for the design of metamaterials for heat conduction^4,5^, mass diffusion^6,7^, and acoustics^8–10^.

Applied to Elasticity, Milton *et al*.^11^ showed that transformation optics produces anisotropic Cosserat materials^12^. Later, Norris and Shuvalov^13^ demonstrated that the transformation method produces Willis materials^14^, having Cosserat materials as simplified particular cases. Considering the little knowledge on the realization of specific Willis or Cosserat materials, Bückman *et al*.^15^ proposed the “direct lattice” transformation approach for the design of realizable mechanical metamaterials from lattices.

As alternative for the design of realizable metamaterials, we proposed the optimization-based approach for metamaterial design^16^. It consists of solving an optimization problem where the objective function measures the accomplishment of the task (e.g., cloaking) assigned to a metamaterial device and the design variables define the distribution of parameters describing the microstructure of the metamaterial in the device. Like the direct-lattice transformation approach^15^, the optimization-based approach directly prescribes how to fabricate the metamaterial at a point, but it excels the former in allowing not only lattices but any quantitatively characterized material.

However, beyond the difficulty yet impossibility of fabricating a specific anisotropic metamaterial, all the above mentioned approaches produce inhomogeneous metamaterials, which is another major deterrent for the design of practical metamaterial devices.

In previous works^17,18^, we introduced the idea of considering a device for heat flux manipulation to behave as a metamaterial as a whole but being made of a few simple even isotropic materials. This rebuts the mainstream belief that it is imperative to use anisotropic inhomogeneous metamaterials for the manipulation of macroscopic fields.

Now, this way of designing what are maybe the easiest to fabricate devices for the manipulation of macroscopic fields is extended to elastostatic problems. Particularly, its capability is proven by means of the design of a device for elastostatic cloaking or unfeelability using only two isotropic elastic materials.

## Methodology

**Figure 1:**
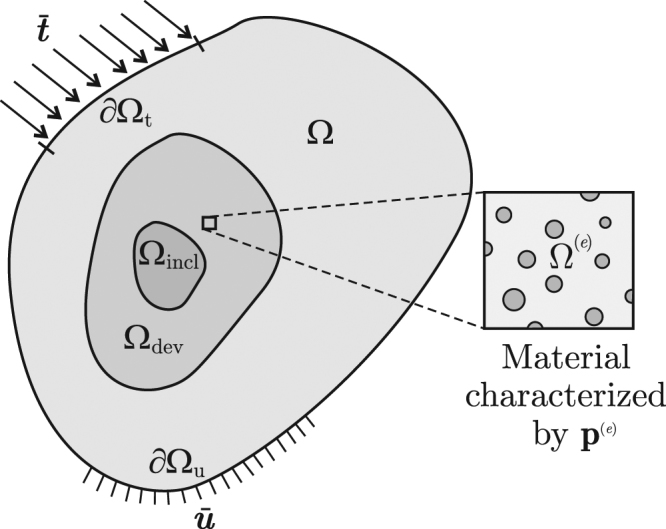
Domain, load and displacement boundary conditions in a body Ω with a inclusion Ω_incl_, to be cloaked using a device Ω_dev_ with non homogeneous microstructure.

Let Ω be originally a body made of a given arbitrary material, and **u**_0_ be the displacement field in Ω under given tractions $\bar{{\rm{t}}}$ and displacements $\bar{{\rm{u}}}$ in the portions ∂Ω_t_ and ∂Ω_u_, respectively, of the boundary ∂Ω of Ω. Then, let us assume that an inclusion of a second material, with either much less or more stiffness than the original one, is embedded in the region Ω_incl_ ⊂ Ω, as shown in Fig. 1. The presence of the inclusion sensibly affects the displacement field in Ω, which is now referred to as **u**.

Using the finite element method (FEM), the displacement **u** at any point **x** ∈ Ω is approximated as follows:

1 $${\bf{u}}({\bf{x}})=\sum _{n=1}^{{N}_{{\rm{n}}{\rm{o}}{\rm{d}}}}{\phi }_{n}({\bf{x}}){{\bf{u}}}_{n}={\boldsymbol{\Phi }}{\bf{U}},$$

where ${\phi }_{n}$ is the shape function associated to the node *n* of the finite element mesh of Ω, *n* = 1, 2, …, *N*_nod_, and **u**_*n*_ is the (unknown) displacement at this node; for ${\bf{u}}\in {{\mathbb{R}}}^{{N}_{{\rm{\dim }}}}$ (*N*_dim_ = 2 for plane strain and plane stress states, *N*_dim_ = 3 for general 3D problems), ${\boldsymbol{\Phi }}\in {{\mathbb{R}}}^{{N}_{{\rm{\dim }}}\times {N}_{{\rm{dof}}}}$ is the matrix grouping the shape functions, *N*_dof_ = *N*_dim_*N*_nod_, and ${\bf{U}}\in {{\mathbb{R}}}^{{N}_{{\rm{dof}}}}$ is the vector of nodal displacements, whose components ***u***_*n*_ = $\bar{{\rm{u}}}$(**x**_*n*_) are prescribed for all the nodes **x**_*n*_ ∈ ∂Ω_u_. On the other hand, the unknown components of **U** are determined as solution of the equilibrium equations:

2 $${\bf{K}}{\bf{U}}={\bf{F}},$$

where **K** and **F** are the global stiffness matrix and the nodal load vector, respectively, given by

3 $${\bf{K}}={\int }_{{\rm{\Omega }}}{{\bf{B}}}^{T}{\bf{CB}}{\rm{d}}{V},$$

4 $${\bf{F}}={\int }_{{\rm{\partial }}{{\rm{\Omega }}}_{t}}{{\boldsymbol{\Phi }}}^{T}\bar{{\bf{t}}}{\rm{d}}S,$$

with **B** as the strain/displacement matrix and **C** as the effective elastic moduli. The linear algegraic system of equations (2) is the FEM version of the equilibrium equations for linear elastic solids, whose solution is widely detailed in the literature (see for instance the book of Zienkiewicz and Taylor on the basics of FEM^19^).

Now, to cloak the inclusion Ω_incl_ requires to have **u** = **u**_0_ at the points located in a certain region Ω_cloak_ where the displacement is to be sensed. To this end, we must design the material inside a certain region Ω_dev_ (from now on, referred to as the “device”) surrounding Ω_incl_. In general, such material *by design* or *metamaterial* has a variable microstructure throughout the device Ω_dev_.

Given Ω divided in finite elements, let the microstructure at each finite element Ω^(*e*)^ ∈ Ω_dev_ be quantitatively characterized by *N*_par_ scalar parameters grouped into the vector **p**^(*e*)^; this means that any effective material property at Ω^(*e*)^ can be expressed as a function of **p**^(*e*)^, as it is the case of the elastic moduli **C** = **C**(**p**^(*e*)^). The microstructure throughout the device is characterized by the vector **P** made of all the parameters **p**^(*e*)^ of all the *N*_dev_ finite elements in the device Ω_dev_, so ${\bf{P}}\in {{\mathbb{R}}}^{{N}_{{\rm{var}}}}$ with *N*_var_ = *N*_par_*N*_dev_. Consequently, being **K** = **K**(**P**) in the equilibrium equations (2), it immediately follows that **u** at any **x** ∈ Ω depends on **P**, i.e. **u** = **u**(**x**, **P**).

Then, the cloaking design problem consists of finding **P** (i.e., the microstructure distribution in Ω_dev_) such that **u**(**x**, **P**) = **u**_0_(**x**) for all **x** ∈ Ω_cloak_. To make such problem amenable to be numerically solved, instead of checking the accomplishment of the cloaking task at all the points **x** ∈ Ω_cloak_, let us do it at *N*_check_ predefined checking points ${\bar{{\bf{X}}}}^{(i)}$ ∈ Ω_cloak_. So, the discrete form of the cloaking design problem can be stated as: to find ${\bf{P}}\in {{\mathbb{R}}}^{{N}_{{\rm{v}}{\rm{a}}{\rm{r}}}}$ such that

5 $${\bf{u}}({\bar{{\bf{x}}}}^{(i)},{\bf{P}})={{\bf{u}}}_{0}({\bar{{\bf{x}}}}^{(i)}),i=1,\ldots ,{N}_{{\rm{check}}}.$$

Further, note that not every ${{\bf{p}}}^{(e)}\in {{\mathbb{R}}}^{{N}_{{\rm{par}}}}$ defines an admissible microstructure, which constrains the search of **P** to a feasible design set ${\mathscr{D}}\subset {{\mathbb{R}}}^{{N}_{{\rm{v}}{\rm{a}}{\rm{r}}}}$. In general, it will not be possible to exactly accomplish the cloaking task (5) by looking for **P** in the set ${\mathscr{D}}$. Therefore, in order to obtain an optimal design, we propose to solve the following nonlinear constrained optimization problem:

6 $$\begin{array}{c}min\\ {\bf{P}}\in {\mathscr{D}}\end{array}\,{f}_{{\rm{o}}{\rm{b}}{\rm{j}}}({\bf{P}}),$$

where **P** plays the role of decision or design variables, *N*_var_ is the number of design variables, and *f*_obj_ is the objective function, defined as

7 $${f}_{{\rm{obj}}}=\sqrt{\frac{1}{{N}_{{\rm{check}}}}\sum _{i\mathrm{=1}}^{{N}_{{\rm{check}}}}{\Vert {\bf{u}}({\bar{{\bf{x}}}}^{(i)},{\bf{P}})-{{\bf{u}}}_{0}({\bar{{\bf{x}}}}^{(i)})\Vert }^{2}},$$

which is the root mean square error (RMSE) in the accomplishment of the cloaking task (5).

Using the current approach, i.e. defining the cloaking task as the optimization problem (6), it is still possible to obtain a design for perfect cloaking if the feasible design set ${\mathscr{D}}$ is rich enough. If not, we will obtain a design for which the error in the accomplishment of the task reaches a minimum.

### Parametrization of the microstructure

In a broad range of materials, the so-called “quantitatively characterized” materials^20^, the effective material properties depend on a few parameters: the thickness and the orientation of layers in laminates^16^, the density and the irregularity factors in materials with isolated inhomogeneities^20^, the size of the prismatic inclusions in an elastic composite^21^, the fiber orientation in fiber-reinforced polymers^22^, the size of particles or beads in coating of dental implants^23^, etc. These parameters are the components of the vector **p**^(*e*)^ at a finite element.

The metamaterials designed using the direct lattice transformation approach^15^ are quantitatively characterized materials where **p**^(*e*)^ defines the topology of the unit cell of the lattice at the element Ω^(*e*)^. Actually, Cosserat materials like those designed using the transformation optics approach can be represented by assuming the components of **p**^(*e*)^ to be the effective material properties (or their components if they are tensors), following the free material optimization (FMO) approach^24^. By this way, the current optimization-based approach for metamaterial design is capable of embodying the previous design approaches as particular cases.

After designing a device for thermal cloaking using variable metamaterial distribution^16^, and realizing the difficulty of fabricating it, we proposed^18^ that the material at any point of the device should be chosen from a list of previously defined candidate materials. This approach, leading to easy-to-make solutions, is adopted in this work.

Then, after adopting a set of candidate materials consisting of *N*_cand_ linear elastic solids with known elastic moduli **C**_*m*_, *m* = 1, 2,…, *N*_cand_, the effective elastic moduli **C**^(*e*)^ at a finite element Ω^(*e*)^ ∈ Ω_dev_ is defined by the mixture law:

8 $${{\bf{C}}}^{(e)}=\sum _{m\mathrm{=1}}^{{N}_{{\rm{cand}}}}{\varphi }_{m}^{(e)}{{\bf{C}}}_{m},$$

where ${\varphi }_{m}^{(e)}$ is the fraction of the candidate material *m* at the finite element Ω^(*e*)^. Since the material at Ω^(*e*)^ must be one of the candidates instead of a mixture of them, ${\varphi }_{m}^{(e)}$ should be either one or zero. This makes (5) an integer optimization problem, which is too expensive to solve when the number of design variables is large (as it is the case when there are *N*_cand_ variables per finite element in a fine enough mesh).

Then, we transform this integer optimization problem into a continuous one, which can be efficiently solved using gradient-based algorithms. To this end, we use the “discrete material optimization” (DMO) technique^25^, where ${\varphi }_{m}^{(e)}$ is defined as a continuous function of the real variables ${\rho }_{m}^{(e)}\in \mathrm{[0},\mathrm{1]}$ as follows:

9 $${\varphi }_{m}^{(e)}=\frac{{({\rho }_{m}^{(e)})}^{p}{\prod }_{k={\rm{1}};k\ne {\rm{m}}}^{{N}_{{\rm{cand}}}}[1-{({\rho }_{k}^{(e)})}^{p}]}{{\rho }_{j}^{(e)}{\sum }_{j=1}^{{N}_{{\rm{chand}}}}{()}^{p}{\prod }_{k={\rm{1}};k\ne j}^{{N}_{{\rm{cand}}}}[1-{({\rho }_{k}^{(e)})}^{p}]}\equiv {\varphi }_{m}({{\bf{p}}}^{(e)}),$$

with ${{\bf{p}}}^{(e)}=[{\rho }_{1}^{(e)},\ldots ,{\rho }_{{N}_{{\rm{cand}}}}^{(e)}]$, and ${\rho }_{m}^{(e)}$ playing now the role of design variables. Note that the continuous optimization problem (6) is subject to the box constraints $0\le {\rho }_{m}^{(e)}\le 1$. Like in topology optimization^26^, intermediate values of the material fraction ${\varphi }_{m}^{(e)}$ are penalized by setting *p* ≥ 3 in (9).

## Results

**Figure 2:**
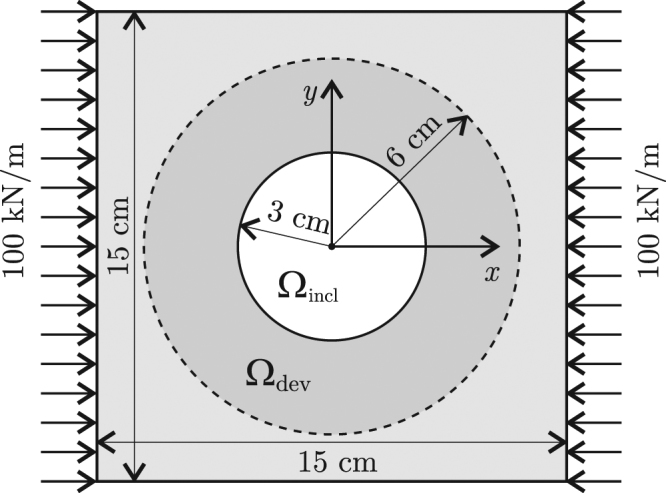
Geometry and load for the example of mechanical cloaking under a single load.

Let us apply the current methodology for the design of a mechanical cloaking device similar to that one designed by Bückmann *et al*.^15^ using the lattice transformation approach. Given a holed plate Ω made of nylon, compressed under the load 100 kN/m applied at two opposite faces (see Fig. 2), the problem consists of cloaking the hole Ω_incl_ using a coating ring that occupies the region Ω_dev_.

As candidate materials for building the cloak, we chose two isotropic materials: aluminum, with Young modulus *E* = 69 GPa and Poisson ration *ν* = 0.32, and polytetrafluoroethylene (PTFE), with *E* = 0.5 GPa and *ν* = 0.4. The stiffness of these materials are sensibly different than that of the base material (nylon has *E* = 3 GPa and *ν* = 0.4).

The plate Ω is assumed to be under plane stress conditions and modeled using a mesh of 200 × 200 bilinear square finite elements.

**Figure 3:**
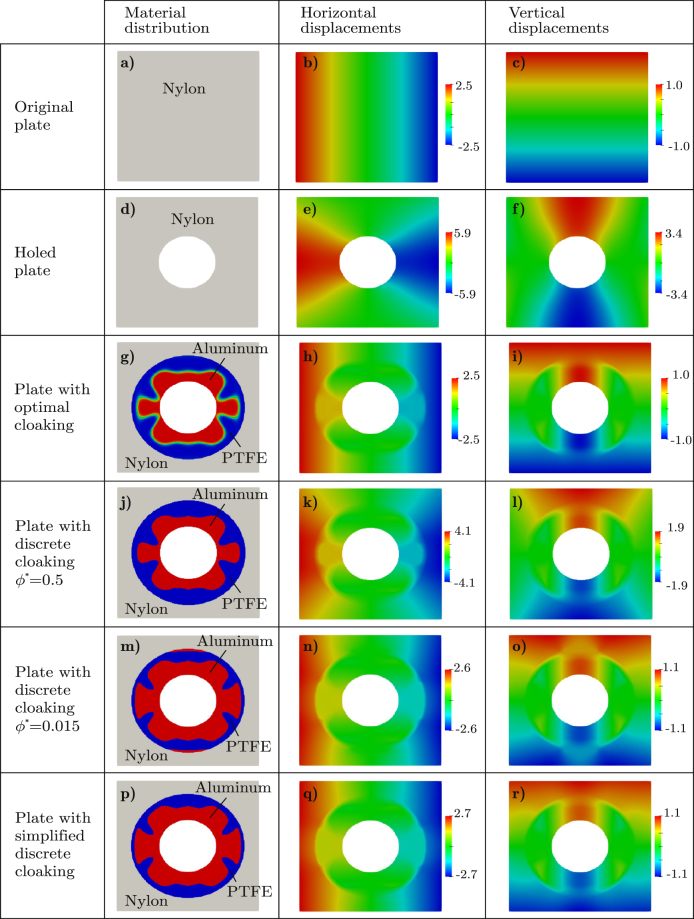
Single load case. Material distribution and displacements for the homogeneous plate without hole, the homogeneous plate with hole and the plate with the cloaked hole; displacements are given in millimeters.

Considering the nylon plate without the hole, the FEM solution for the displacement field, that is **u**_0_, is that given in Fig. 3(b,c).

For the nylon plate with the hole Ω_incl_, let us take the previous mesh and then discard the finite elements whose centers lie in Ω_incl_. In this case, the FEM solution for the displacement is that shown in Fig. 3(e,f), and the error in the accomplishment of the cloaking task is ${{\rm{R}}{\rm{M}}{\rm{S}}{\rm{E}}}_{{\rm{n}}{\rm{o}}{\rm{c}}{\rm{l}}{\rm{o}}{\rm{a}}{\rm{k}}}=1.845\,{\rm{m}}{\rm{m}}=0.685\,{\rm{m}}{\rm{a}}{\rm{x}}\parallel {{\bf{u}}}_{0}\parallel$ , with ${\rm{m}}{\rm{a}}{\rm{x}}\parallel {{\bf{u}}}_{0}\parallel =2.693\,{\rm{m}}{\rm{m}}$.

Now, let us design a device Ω_dev_ around the hole Ω_incl_ such that the displacements approach **u**_0_ at all the *N*_check_ = 19972 nodes outside Ω_dev_. Using the current approach, we seek to accomplish the cloaking task with a minimum error by solving the optimization problem (6) with the design variables **P** defining the material at all the *N*_dev_ = 15084 finite elements whose centers lie in Ω_dev_. So, the total number of design variables is *N*_var_ = 2*N*_dev_ = 30168. In this work, this large nonlinear constrained optimization problem (6) is solved using the interior-point algorithm known as IPOPT^27^. As suggested by Sigmund^28^, a smoothed Heaviside density filter was applied to reduce both checker-board type instabilities and “grey zones” (those where the material is none of the candidates but a composite of them).

Figure 3(g) shows the so-computed optimal material distribution in the cloaking device, giving the displacement field depicted in Fig. 3(h,i), with RMSE = 0.00417RMSE_nocloak_.

To eliminate the grey zones that are observed in the optimal design shown in Fig. 3(g), let us apply the simple a posteriori black-and-white filter proposed by Fachinotti *et al*.^17^: if the aluminum fraction at an element is greater than a certain threshold ϕ^*^ ∈ (0, 1), the element is assumed to be fully made of aluminum; otherwise, it is made of PTFE. By this way, the device becomes easier to fabricate at the expense of deteriorating the accomplishment of the cloaking task since the material distribution is not longer optimal. A priori, the natural choice for ϕ^*^ should be ϕ^*^ = 0.5, that is equivalent to assume that the material at an element is that having the highest fraction; however, the corresponding device (Fig. 3(j)) poorly performs the cloaking task, with RMSE = 0.5495RMSE_nocloak_. Actually, the lowest error in the cloaking task is achieved for the quite low threshold ϕ^*^ = 0.015, giving raise to the device shown in Fig. 3(m), for which RMSE = 0.0610RMSE_nocloak_.

For easier fabrication, let us eliminate the narrow aluminum areas at the top and at the bottom of the device shown in Fig. 3(m), obtaining that shown in Fig. 3(p), for which RMSE = 0.0896RMSE_nocloak_. This device has an aluminum core with a variable thick coat of PTFE, the whole acting as a compliant mechanism to cloak the hole.

Considering the measure of the error in the task accomplishment as defined by Bückmann *et al*.^15^, that is

10 $${\rm{\Delta }}=\frac{\sqrt{\sum _{i=1}^{{N}_{{\rm{check}}}}{\Vert {\bf{u}}({\bar{{\bf{x}}}}^{(i)},{\bf{P}})-{{\bf{u}}}_{0}({\bar{{\bf{x}}}}^{(i)})\Vert }^{2}}}{\sqrt{\sum _{i\mathrm{=1}}^{{N}_{{\rm{check}}}}{\Vert {{\bf{u}}}_{0}({\bar{{\bf{x}}}}^{(i)})\Vert }^{2}}},$$

we obtain Δ = 0.40% for the optimal device (that of Fig. 3(g)) and Δ = 8.56% for the simplest device (that of Fig. 3(p)). This is a very good cloaking performance compared to that obtained by Bückmann *et al*.^15^, for whom Δ ≈ 20%.

### Extension to multiple loads

This methodology can be easily applied to accomplish another tasks by just changing the objective function. Further, to change the involved geometries (either that of the device, the inclusion or the body where it is embedded) or the boundaries conditions (loads and displacements) only implies to modify the finite element model, without altering to any extent the current optimization-based design procedure. So, complicated, maybe three-dimensional, real industrial or engineering problems can be straightforwardly accounted for.

For instance, to account for multiple loads implies an easy redefinition of the objective function. Let **u**_0_ = **u**_0_(**x**, **F**^(*α*)^) be the displacement caused by the external force **F**^(*α*)^ in the domain Ω without the inclusion, and **u** = **u**(**x**, **P**, **F**^(*α*)^) be the displacement caused by that force in Ω in presence of the inclusion Ω_incl_ together with the cloaking device occupying Ω_dev_, where the material distribution is defined by **P**. In presence of multiple loads ${{\bf{F}}}^{\mathrm{(1)}},{{\bf{F}}}^{\mathrm{(2)}},\ldots ,{{\bf{F}}}^{({N}_{{\rm{load}}})}$, the objective function representing the error in the accomplishment of the global cloaking task can be defined as the weighted sum of the RMSEs in the accomplishment of the individual tasks, that is

11 $${f}_{{\rm{obj}}}=\sum _{\alpha =1}^{{N}_{{\rm{load}}}}{\omega }_{\alpha }\sqrt{\frac{1}{{N}_{{\rm{check}}}}\sum _{i=1}^{{N}_{{\rm{check}}}}{\Vert {\bf{u}}({\bar{{\bf{x}}}}^{(i)},{\bf{P}},{{\bf{F}}}^{(\alpha )})-{{\bf{u}}}_{0}({\bar{{\bf{x}}}}^{(i)},{{\bf{F}}}^{(\alpha )})\Vert }^{2}},$$

where *ω*_*α*_ is the weight assigned to the task accomplishment for the external force **F**^(*α*)^; typically, *ω*_*α*_ = 1/*N*_load_.

**Figure 4:**
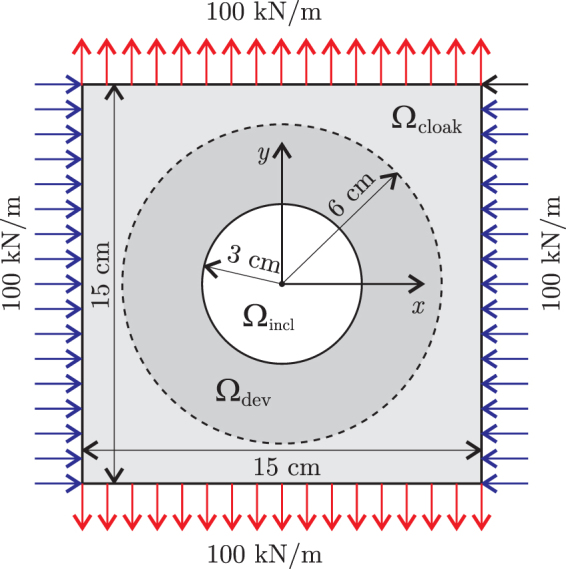
Geometry and load for the example of mechanical cloaking under two loads.

As an application example, let us consider the problem described in the previous section when a vertical tension is added, as shown in Fig. 4. Now, cloaking has to be achieved for horizontal compression (as in the previous example) as well as for vertical tension. In the holed plate fully made of nylon, i.e., without a cloaking device, the error in the accomplishment of the global task is ${f}_{{\rm{obj}}}={f}_{{\rm{nocloak}}}=1.8493{\rm{mm}}=0.6868\,{\rm{\max }}\Vert {{\bf{u}}}_{0}\Vert$, with ${{\rm{RMSE}}}_{{\rm{nocloak}}}^{{\rm{hcomp}}}=$ $0.6853\,{\rm{\max }}\Vert {{\bf{u}}}_{0}\Vert$ for the horizontal compression and ${{\rm{R}}{\rm{M}}{\rm{S}}{\rm{E}}}_{{\rm{n}}{\rm{o}}{\rm{c}}{\rm{l}}{\rm{o}}{\rm{a}}{\rm{k}}}^{{\rm{v}}{\rm{t}}{\rm{e}}{\rm{n}}{\rm{s}}}=0.6853\,max\parallel {{\bf{u}}}_{0}\parallel$ for the vertical tension.

**Figure 5:**
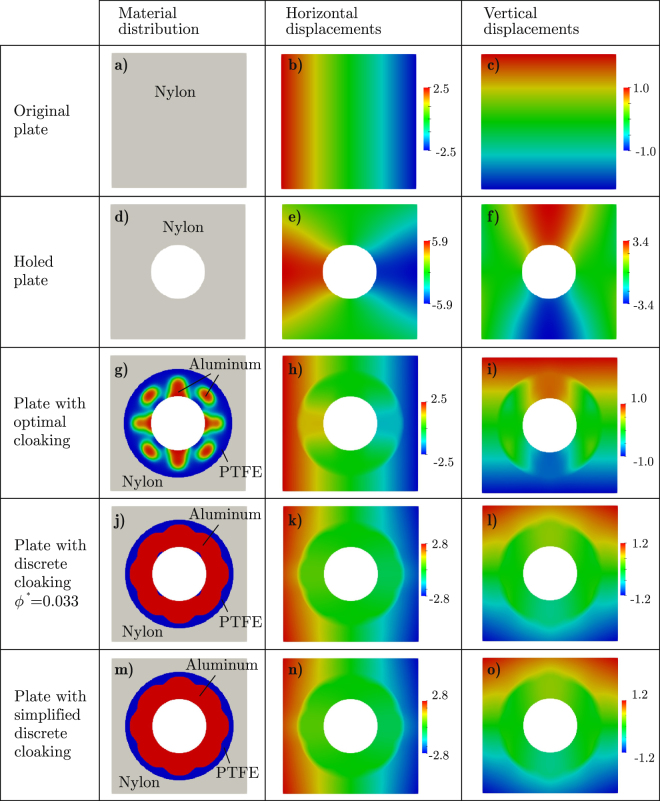
Multiple load case. Material distribution and displacements under horizontal compression for the homogeneous plate without hole, the homogeneous plate with hole and the plate with the cloaked hole; displacements are given in millimeters.

The optimal device is that shown in Fig. 5(g), for which *f*_obj_ = 0.00212*f*_nocloak_, ${{\rm{RMSE}}}^{{\rm{hcomp}}}=0.00207{{\rm{RMSE}}}_{{\rm{nocloak}}}^{{\rm{hcomp}}}$, and ${{\rm{RMSE}}}^{{\rm{vtens}}}=0.00219\,{{\rm{RMSE}}}_{{\rm{nocloak}}}^{{\rm{vtens}}}$. Although this device accomplished the cloaking task very well, it is severely affected by grey zones.

If we define a fully discrete device by assuming that those finite elements in the optimal device having an aluminum fraction greater than ϕ^*^ are completely made of aluminum, the best design is that obtained for ϕ^*^ = 0.033, shown in Fig. 5(j), for which the global error is *f*_obj_ = 0.1101*f*_nocloak_, and the individual errors are ${{\rm{RMSE}}}^{{\rm{hcomp}}}=0.1120\,{{\rm{RMSE}}}_{{\rm{nocloak}}}^{{\rm{hcomp}}}$ and ${{\rm{RMSE}}}^{{\rm{vtens}}}=0.1081\,{{\rm{RMSE}}}_{{\rm{nocloak}}}^{{\rm{vtens}}}$.

If, for easier manufacturability, we eliminate from this device those little PTFE areas adjacent to the hole obtaining the device shown in Fig. 5(m), the accomplishment of the task is slightly affected, with *f*_obj_ = 0.1104*f*_nocloak_, ${{\rm{RMSE}}}^{{\rm{hcomp}}}=0.1124\,{{\rm{RMSE}}}_{{\rm{nocloak}}}^{{\rm{hcomp}}}$, and ${{\rm{RMSE}}}^{{\rm{vtens}}}=0.1084\,{{\rm{RMSE}}}_{{\rm{nocloak}}}^{{\rm{vtens}}}$.

Let us remark that, because of the highly non-discrete nature of the optimal device (Fig. 5(g)), the accomplishment of the cloaking task is considerably affected by the black-and-white filtering. However, this is still satisfactory as it can be realized when computing the error Δ in the cloaking task as defined by Bückmann *et al*.^15^ (see equation (10)): Δ = 10.74% for cloaking the horizontal compression, and Δ = 10.40% for cloaking the vertical tension.

Note that the displacements shown in Fig. 5 are those produced by the horizontal compression, say **u**^hcomp^. Since the devices (the optimal as well as the discrete ones) are practically symmetric with respect to the center, the displacements produced by the vertical tension, say **u**^vtens^, are easily derivable from **u**^hcomp^: actually, the horizontal and vertical components of **u**^vtens^ have color maps almost identical to those shown in Fig. 5 but their thresholds are interchanged. Not surprisingly, the current devices accomplish the cloaking task almost equally for both loads.

It is also not surprising, and worth noting, that a device designed for multiple loads performs the cloaking task for each individual load poorly than the device specifically designed for such load, as done in the previous section.
